# Optimal indications of endocrine therapy alone as adjuvant systemic treatment of breast cancer

**DOI:** 10.1038/sj.bjc.6603916

**Published:** 2007-08-28

**Authors:** R Horii, F Akiyama, Y Ito, T Iwase

**Affiliations:** 1 Department of Pathology, The Cancer Institute of the Japanese Foundation for Cancer Research, Tokyo, Japan; 2 Department of Breast Oncology, The Cancer Institute Hospital of the Japanese Foundation for Cancer Research, Tokyo, Japan

**Keywords:** breast cancer, adjuvant therapy, St Gallen expert consensus meeting, pathology

## Abstract

We examined the validity of the St Gallen algorithm for Japanese breast cancer patients and sought the optimal indications of endocrine monotherapy as adjuvant systemic treatment. According to the 2005 St Gallen algorithm, endocrine responsiveness (responsive, uncertain, or non-responsive) and recurrence risk (low, intermediate, or high) were assessed in 436 invasive breast cancer patients, who underwent surgery and adjuvant therapy of tamoxifen alone in 1982–1993. Furthermore, intermediate-risk patients were divided into three groups based on lymph node metastasis and number of risk factors as follows: Group A, negative lymph node metastasis and one risk factor; Group B, negative lymph node metastasis and two to five risk factors; and Group C, positive lymph node metastasis. Cumulative 10-year recurrence-free survival (RFS) rates of each type were calculated. Recurrence-free survival was as follows: endocrine responsiveness; responsive: 86.0%, uncertain: 79.5%, non-responsive: 72.4%, risk category; low: 93.3%, intermediate: 84.0%, high: 59.6%, intermediate-risk patients; Group A: 93.5%, Group B: 88.2%, and Group C: 75.0%. In conclusion, patient classification based on St Gallen algorithm appears valid in Japanese breast cancer patients. Endocrine monotherapy may be sufficient as adjuvant treatment in the intermediate-risk patients, in which only one risk factor was present without any metastatic involvement in lymph node.

The algorithm for adjuvant systemic therapy proposed by St Gallen conferences is widely applied in Japan to plan treatments for breast cancer patients. According to St Gallen guidelines, endocrine monotherapy is recommended as adjuvant systemic treatment for low-risk patients and intermediate-risk patients with endocrine responsive tumours. Three therapeutic options, that is endocrine monotherapy, chemotherapy with endocrine therapy, and Trastuzumab, are applicable for intermediate-risk patients with endocrine responsive tumours; however, it remains obscure which option is effective in individual cases. In intermediate-risk patients with endocrine response uncertain tumours, there may be some cases that are good indications of endocrine monotherapy. To identify the optimum indication of endocrine monotherapy as adjuvant systemic treatment for breast cancer, we examined the long-term outcome of Japanese breast cancer patients according to the St Gallen algorithm.

## PATIENTS AND METHODS

A total of 5763 primary breast cancer patients underwent surgery at our institute from January 1982 to December 1993. Out of these patients, postoperative tamoxifen monotherapy was adopted for 659 patients. From these 659 patients, those with the following conditions were excluded: non-invasive cancer; microinvasive cancer; stage IV cancer; male patients; bilateral breast cancer; no residual cancer in surgical material after biopsy; <6 months of tamoxifen therapy; and death by other disease. The remaining 436 patients served as subjects in this study. Median duration of follow-up period was 154 months (range: 120–260 months).

In each subject, tissue sections representative of the primary lesion were embedded in paraffin, stained using haematoxylin and eosin, and subjected to immunohistochemical staining for oestrogen receptor (ER), progesterone receptor (PgR), and HER2. According to the 2005 St Gallen guidelines (Goldhirsch *et al*, 2005), endocrine responsiveness (responsive, uncertain, or non-responsive) and recurrence risk (low, intermediate, or high) were assessed. Furthermore, the intermediate risk group was subdivided into three groups based on lymph node metastasis and number of risk factors as follows: Group A, negative lymph node metastasis and one risk factor; Group B, negative lymph node metastasis and 2–5 risk factors; and Group C, positive lymph node metastasis. Cumulative disease-free survival rates were calculated and compared.

### Immunostaining

Oestrogen receptor and PgR were immunologically stained using ER 1D5 and PgR PgR636 antibodies (DAKO Corp., Carpinteria, CA, USA) and an automatic immunological staining machine. An ENVISION+ kit (DAKO Corp.) was used as a detection system. For ER and PgR assessment, irrespective of stain intensity, positive reactions were defined as staining in ⩾10% of cells.

HER2 was immunologically stained using anti-human HER2/neu rabbit polyclonal antibody and an automatic immunological staining machine. The Hercep Test (DAKO Corp.) was used as a detection system. For HER2 assessment, staining was classified in four grades (0, 1+, 2+, or 3+) according to the Trastuzumab Pathological Committee standards, and positive reactions were defined as 2+ or 3+.

### Statistical analysis

Cumulative recurrence-free survival (RFS) rates were calculated by Kaplan–Meier estimation and compared using log-rank testing. Values of *P*<0.05 were considered statistically significant. Analyses were conducted using STATISTICA (Stat Soft Japan, Tokyo, Japan).

## RESULTS

### Patient characteristics

The patient characteristics are summarised in Table 1. Mean age was 55±9.7 years (range: 23–81 years) and mean tumour diameter was 2.63±1.2 cm (range: non-palpable to 9.0 cm). While 152 patients were premenopausal (35%), 284 patients were postmenopausal (65%). Mean duration of tamoxifen administration was 34.7±19.3 months (range: 6–105 months), and tamoxifen therapy lasted for ⩾24 months in 384 patients (88%).

### Definition of endocrine responsiveness

The concept of ‘endocrine response uncertain’ was first proposed at the 2005 St Gallen consensus meeting. That represents tumour with uncertain responsiveness to endocrine therapy; however, the definition has never been clarified. While the consensus report clearly defines responsive tumours as ER(+)/PgR(+), tumours with uncertain responsiveness as ER(+)/PgR(−), and non-responsive tumours as ER(−)/PgR(−), the classification of ER(−)/PgR(+) tumours has remained unclear (Goldhirsch *et al*, 2005). In the present study, we assessed the outcome of all ER and PgR combinations to establish definitions of the three types of endocrine responsiveness.

ER(+)/PgR(+) status was identified in 210 patients (48%) with 5- and 10-year RFS rates of 89.5 and 84.7% respectively. ER(−)/PgR(+) status was present in 20 patients (5%) with 5- and 10-year RFS rates of 100% each. ER(+)/PgR(−) status was identified in 148 patients (34%) with 5- and 10-year RFS rates of 86.4 and 79.5% respectively. ER(−)/PgR(−) was identified in 58 patients (13%) with 5- and 10-year RFS rates of 72.4% each. ER(−)/PgR(+) patients were thus classified as belonging to the responsive type in the present study.

### Endocrine responsiveness

**Figure 1:**
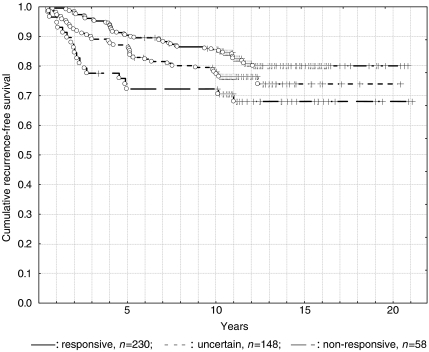
Endocrine responsiveness and long-term outcome.

Based on the above-mentioned classification, a total of 230 patients (53%) were identified as responsive, with 5- and 10-year RFS rates of 90.4 and 86.0% respectively (Figure 1). Another 148 patients (34%) were classified as uncertain, with 5- and 10-year RFS rates of 86.4 and 79.5% respectively. The remaining 58 patients (13%) were non-responsive, with 5- and 10-year RFS rates of 72.4% each. While no significant differences existed between responsive and uncertain groups (*P*=0.09) or between uncertain and non-responsive groups (*P*=0.25), there was a significant difference observed between the responsive and non-responsive groups (*P*=0.01).

### Risk category

**Figure 2:**
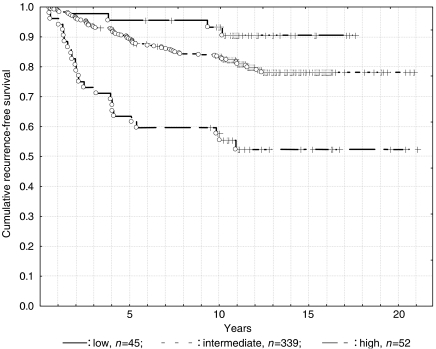
Risk category and long-term outcome.

Risk category was as follows: low risk in 45 patients (10%), with 5- and 10-year RFS rates of 95.6 and 93.3% respectively; intermediate risk in 339 patients (78%) with 5- and 10-year RFS rates of 89.1 and 84.0% respectively; and high risk in 52 patients (12%) with 5- and 10-year RFS rates of 63.5 and 59.6% respectively (Figure 2). While no significant differences were noted between low- and intermediate-risk groups (*P*=0.09), significant differences were observed between low- and high-risk groups (*P*=0.00005) and between intermediate- and high-risk groups (*P*=0.00001).

### Endocrine responsiveness and risk category

Table 2 shows case number, case distribution, and 10-year cumulative RFS rate in relation to endocrine responsiveness and risk category. ten-year RFS rates of 90% were achieved in all low-risk patients.

### Intermediate-risk patients

**Figure 3:**
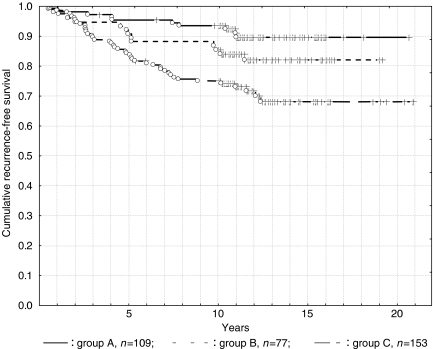
Intermediate-risk patients.

Group A comprised 109 patients (32%) with 5- and 10-year RFS rates of 95.4 and 93.5% respectively (Figure 3). Group B included 77 patients (23%) with 5- and 10-year RFS rates of 90.8 and 88.2% respectively. Group C had 153 patients (45%) with 5- and 10-year RFS rates of 84.3 and 75.0% respectively. While no significant differences were seen between Groups A and B (*P*=0.12) or between Groups B and C (*P*=0.05), a significant difference was identified between Groups A and C (*P*=0.0001).

Table 3 shows case number, case distribution, and 10-year cumulative RFS rate in relation to endocrine responsiveness. Among the intermediate-risk patients, RFS rates of 90% were achieved in all Group A and Group B patients with endocrine responsive tumours.

## DISCUSSION

Compared to the 2003 St Gallen guidelines, the 2005 guidelines include two major revisions to patient classification (Goldhirsch *et al*, 2003, 2005). These revisions are inherited in 2007 consensus conference (Goldhirsch *et al*, 2007). First revision is that endocrine responsiveness is divided into responsive, non-responsive, and uncertain classifications. ‘Endocrine response uncertain’ is defined as some expression of hormone receptors either quantitatively low or qualitatively insufficient to indicate substantial chance for response to endocrine therapies alone. However, whether all patients of this category need for chemotherapy or not is a matter for argument. Second revision is that lymph node involvement is one of the factors for risk categorisation, and risks are classified into three grades: low, intermediate, and high. Therefore, intermediate-risk patients accounted for large population of breast cancer cases, and several therapeutic options are available for these patients. However, in St Gallen guidelines, there are no clear statements how to select these options for individual patients.

A long-term outcome of Japanese breast cancer patients is known as better than that of Western patients (Sakamoto *et al*, 1979). Whether the consensuses reached in Western countries are applicable to Japanese breast cancer patients must be ascertained.

As to endocrine responsiveness, the outcome of unclassified ER(−)/PgR(+) was assessed, and each type of endocrine responsiveness was defined in the present study. While relatively few ER(−)/PgR(+) cases were encountered, prognosis was favourable. The present study thus classified ER(−)/PgR(+) tumours as belonging to responsive tumours.

Subjects were classified based on the above study findings and the 2005 St Gallen guidelines. In terms of endocrine responsiveness, prognosis was most favourable with responsive tumours, followed by uncertain and non-responsive in that order. In terms of risk categories, prognosis was most favourable with low-risk tumours, followed by intermediate- and high-risk, in that order. This confirmed that patient classification based on the 2005 St Gallen guidelines remains valid for Japanese breast cancer patients.

In the present study, intermediate-risk patients accounted for 78% of the total subject population. We, therefore, thought that subdivision of these patients based on lymph node metastasis and number of risk factors was warranted. Prognosis was most favourable with Group A (negative lymph node metastasis and only one risk factor), followed by Group B (negative lymph node metastasis and two to five risk factors) and Group C (positive lymph node metastasis), in that order. Lymph node metastasis was officially added to the 2005 St Gallen guidelines as one of the risk factors, and the significance of this factor as the most important prognostic factor was reaffirmed.

According to the 2005 St Gallen guidelines, either endocrine monotherapy or no therapy is recommended as adjuvant therapy for low-risk patients. In the present study, favourable long-term results were obtained with postoperative tamoxifen monotherapy in low-risk patients, regardless of endocrine responsiveness. This confirmed the validity of the recommended therapy for low-risk breast cancer patients in Japan. Furthermore, compared to low-risk patients, 10-year RFS was comparable for intermediate-risk patients with negative lymph node metastasis and only one risk factor or intermediate-risk patients with negative lymph node metastasis, 2–5 risk factors, and responsive tumours. Postoperative endocrine monotherapy appears sufficient in such intermediate-risk patients. On the other hand, in intermediate-risk patients with positive lymph node metastasis, unfavourable long-term outcomes were obtained with postoperative tamoxifen monotherapy. This type of intermediate-risk patients should receive chemotherapy in addition to endocrine therapy as adjuvant systemic treatment.

In conclusion, patient classifications according to the 2005 St Gallen guidelines were shown to be valid in Japanese breast cancer patients. Based on lymph node metastasis and number of risk factors, it is possible to predict the outcome of each intermediate-risk patient. This point must be kept in mind when choosing the drugs for adjuvant systemic therapy.

## Figures and Tables

**Table 1:** Patient characteristics

	No (%) of patients
*Age*	
<35 years	6 (1)
⩾35 years	430 (99)
	
*Pathological tumour size*	
⩽2 cm	171 (39)
>2 cm	265 (61)
	
*Grade*	
1	188 (43)
2	154 (35)
3	94 (22)
	
*Peritumoral vascular invasion*	
Positive	72 (17)
Negative	364 (83)
	
*ER*	
Positive	358 (82)
Negative	78 (18)
	
*PgR*	
Positive	230 (53)
Negative	206 (47)
	
*HER2*	
Positive (immunohistochemistry: 2+, 3+)	33 (8)
Negative (immunohistochemistry: 0, 1+)	403 (92)
Lymph nodes metastasis	
Negative	231 (53)
Positive (1–3 involved nodes)	166 (38)
Positive (4 or more involved nodes)	39 (9)

ER=oestrogen receptor; PgR=progesterone receptor.

**Table 2:** Endocrine responsiveness/risk category and cumulative recurrence-free survival (RFS)

	**Responsive**	**Uncertain**	**Non-responsive**
*Low*			
No. of cases	29	16	NA
Case distribution	7%	4%	
10-year RFS	93.0%	93.8%	
			
*Intermediate*			
No. of cases	176	113	50
Case distribution	40%	26%	11%
10-year RFS	88.0%	81.2%	75.9%
			
*High*			
No. of cases	25	19	8
Case distribution	6%	4%	2%
10-year RFS	64.0%	57.9%	50.0%

NA=not applicable.

**Table 3:** Endocrine responsiveness and cumulative recurrence-free survival (RFS) in intermediate risk patients

	**Responsive**	**Uncertain**	**Non-responsive**
*A*			
No. of cases	59	35	15
Case distribution	17%	10%	5%
10-year RFS	91.5%	94.3%	100%
			
*B*			
No. of cases	32	25	20
Case distribution	9%	7%	6%
10-year RFS	93.8%	87.3%	75.0%
			
*C*			
No. of cases	85	53	15
Case distribution	25%	16%	5%
10-year RFS	83.5%	65.1%	53.3%
